# *Helicobacter pylori* Infection and Light Chain Gammopathy

**DOI:** 10.1155/2013/348562

**Published:** 2013-12-02

**Authors:** José A. Girón, Shawn L. Shah

**Affiliations:** ^1^Florida State University College of Medicine, Tallahassee, FL 32306, USA; ^2^University of Central Florida College of Medicine, Orlando, FL 32816, USA; ^3^Department of Medicine, Orlando Regional Medical Center, Orlando, FL 32806, USA; ^4^Department of Medicine, Dartmouth-Hitchcock Medical Center, Lebanon, NH 03755, USA

## Abstract

*Objective. Helicobacter pylori* provokes a host of immune alterations upon colonizing the gastric mucosa. *Design.* We report 22 individuals with confirmed *Helicobacter pylori* infection who were also managed for the concurrent elevation of immunoglobulin free light chain (kappa and lambda) levels. *Result.* Of the 22 patients, 15 patients (68.2%) had elevated free light chain levels: 6 patients (40%) had only kappa chain elevation, 2 patients (13.3%) had only lambda chain elevation, and 7 patients (46.7%) had both kappa and lambda chain elevation. Twenty out of the 22 patients (90.9%) were microbiologically confirmed cured with 3 patients being lost to follow-up for repeat levels. Of the 3 patients who were lost to follow-up, 1 patient had only kappa chain elevation, 1 patient had only lambda chain elevation, and 1 patient had both kappa and lambda chain elevation. For those who were cured (19 patients), 5 patients with kappa elevation had normalized values, 4 patients with lambda elevation had normalized values, and 2 patients with combined kappa and lambda elevation had normalized values. For 6 out of the 19 patients, the light chain levels remained elevated. *Conclusion.* We speculate that the *Helicobacter pylori* infection disrupts the immunoglobulin system with potential implications being discussed below.

## 1. Introduction

*Helicobacter pylori* is one of the most pervasive bacterial pathogens worldwide and is associated with an increased risk of gastritis, gastric and duodenal ulcers, gastric adenocarcinoma, and gastric mucosa-associated lymphoid tissue (MALT) lymphoma [1, 2]. Indeed, it has been nearly three decades since the Australian physicians Warren and Marshall first isolated the pathogen and elucidated its association with gastric and duodenal ulcers as well as gastritis, leading to significant research towards the treatment of these gastrointestinal disorders [3]. While the incidence of the* H. pylori* infection varies largely by socioeconomic status, geographic region, age, and race, approximately one-half of the world's population has been estimated to be infected, with the prevalence ranging from 30 to 40 percent in the United States [4, 5]. In fact, in the United States, the prevalence increases from less than 20% at 20 years of age to nearly 50% at 50 years of age [6]. Today, with the advent of the newer concomitant quadruple therapy, the eradication of *H. pylori* has experienced success rates greater than 90 percent [7, 8].

In a patient with light chain deposition disease, a transient increase in light chain levels was observed upon *H. pylori* infection; while levels did not normalize after microbiological cure, they down trended and returned to the patient's baseline. The aforementioned phenomenon led us to evaluate if there was in fact a disruption in the immunoglobulin system, specifically in regards to free light chains, that accompanies the *Helicobacter pylori* infection (J. Girón, personal observation).

## 2. Methods

In total, twenty-two adult patients—7 female patients and 15 male patients—who were diagnosed and selected randomly with *H. pylori* disease from 2009 to 2011, confirmed by a positive stool antigen test *via* an enzyme immunoassay (Quest or LabCorp), had a quantitative evaluation of free kappa and lambda light chain levels in serum (Table 1). The noninvasive *H. pylori *stool antigen test that was utilized has an overall sensitivity of 91% and specificity of 93% [9]. All of the patients were subject to a thorough history and clinical examination in addition to a complete laboratory investigation to determine their *H. pylori* status as well as the presence of other potential infectious diseases.

The patients typically received a 7-to-10 day oral regimen of clarithromycin, metronidazole, omeprazole, and bismuth subsalicylate (Table 3). All of the patients with hepatitis C, except patient 19, received *Helicobacter pylori* treatment prior to the institution of treatment for hepatitis C. Thus, changes in light chain levels were recorded before the patients were treated for hepatitis C. Patient 19 received treatment for strongyloidiasis but did not adhere to treatment for *H. pylori* or hepatitis C (Table 4(c)). Confirmation of a microbiological cure of the *H. pylori* infection with a repeat stool antigen test was performed along with a repeat measurement of the free light chain levels if previously elevated. All patients were informed of the additional blood testing. Confirmatory testing was performed from 3 weeks to 130 weeks after the completion of treatment. The associated comorbid conditions were also recorded (Table 2).

## 3. Results

The study included 22 patients with ages ranging from 30 to 85 years (mean of 51.9 years of age) (Table 1). Fifteen patients had confirmed *H. pylori* disease and elevated light chain levels. For 6 out of 15 patients (40%), only elevated free kappa light chain levels were found, and for 2 out of 15 patients (13.3%), only elevated free lambda light chain levels were found. For 7 out of 15 patients (46.7%), both kappa and lambda light chain elevation was found. Overall, 15 out of the 22 patients (68.2%) had an elevation in free kappa and/or lambda light chain levels (Tables 2 and 3), while 7 patients (31.8%) had normal lambda and kappa light chain levels.

Twenty of the twenty-two patients (90.9%) were confirmed microbiologically cured of the *H. pylori* infection as evidenced by a repeat stool antigen test (Table 1). Three patients (13.6%) failed to follow-up in an outpatient setting to repeat free light chain values. Out of the 3 patients who were lost to follow-up for repeat light chain values, 1 patient had only kappa chain elevation, 1 patient had only lambda chain elevation, and 1 patient had both kappa and lambda chain elevation.

We had 15 patients with confirmed *H. pylori* eradication and abnormal light chain levels. Light chain levels were monitored over a period of 3 to 130 weeks. Of these 15 patients, 3 patients were lost to follow-up (Table 4(d)). Three patients with solitary kappa light chain elevation experienced normalization of levels. One patient with solitary lambda light chain elevation experienced normalization of levels. Finally, 2 patients with combined kappa and lambda light chain elevation experienced normalization of both levels (Tables 4(a), 4(b), and 4(c)). The remaining 6 patients had persistent elevation of kappa and/or lambda light chain levels after successful eradication of *H. pylori* (Table 4(c)).

We had 7 patients with hepatitis C and *H. pylori* infection. In 5 patients tested for cryoglobulins, all 5 were negative.

## 4. Discussion

To our knowledge, this is the first observation of an association between *Helicobacter pylori* infection and an elevation of immunoglobulin free light chains. We found that 15 out of 22 patients (68.2%) had at least one light chain elevated at the time of *H. pylori* diagnosis, while 7 out of 22 (31.8%) patients had no disturbance in light chain levels. Our study has not investigated the cause of this phenomenon. We speculate that either unique bacterial strain factors, a host genetic difference among patients, or a combination of both might account for our observations. Some strains of *H. pylori* have been identified as having low virulence and fail to incite any gastroduodenal disease. Certain hosts carry a particular HLA-DQA gene which induces a potent *H. pylori* antibodies and T-cell response [10–13]. Our findings raise the possibility of stratifying *H. pylori* patients into those with abnormal light chain levels and those with normal levels. Long term follow-up of patients with persistent elevation of light chain levels may lead to a better understanding of the biologic processes involved.

A few reports of Russell body gastritis in *H. pylori* patients show light chain deposits in the tissues of the gastric mucosa and raise an intriguing notion as to a possible unique immunologic defense mechanism or immune disruption occurring in *H. pylori* infected tissue [14–16]. For example, while *H. pylori*'s urease activity is vital for its survival, an enhanced immunological response to the enzyme may decrease the bacteria's persistence and sequelae [17]. The gene products of cytotoxin-associated gene A (CagA) and vacuolating cytotoxin A (VacA) are associated with a robust production of cytokines and tissue inflammation [18, 19]. The presence or absence of the aforementioned proteins assists in classifying the distinct strains of *H. pylori* as well as providing further information as to its association with disease [20]. The virulent *cag *pathogenicity island (PAI), which encodes the bacterial oncoprotein CagA, has been strongly linked to carcinogenesis and is found in nearly 70% of *H. pylori* strains worldwide [21, 22]. The connection between *H. pylori* and disease may lie in the epigenetic control of gene expression as the CagA protein not only interferes with the host's cytoskeleton and cell-signaling pathways but also reduces levels of *p53* expression and increases the persistence of damaged DNA [23, 24]. Furthermore, the CagA protein has been shown to disrupt typical epithelial differentiation similar to the process of epithelial-to-mesenchymal transition (EMT) and is thought to play a role in tumorigenesis [25]. Thus, it has been theorized that gastric carcinomas can be mediated by *Helicobacter pylori*'s dynamic inflammatory effects on the *p53* tumor suppressor gene, which has been found in 38–71% of gastric neoplasms [26, 27]. Certainly, further investigation of the gastric and intestinal flora along with the epigenetic influence of *cagPAI* may provide insight into how *Helicobacter pylori* is influenced by its gastrointestinal environment and can incite immune-mediated disease in patients [28].

Terrier et al. (2009) have reported elevated immunoglobulin light chain levels in patients with hepatitis C infection and mixed cryoglobulinemia [29]. In this series, 5 of the 7 patients with hepatitis C in whom cryoglobulin levels were tested had negative values, suggesting that the elevation of light chain levels was not associated with cryoglobulinemia.

Except patient 19, all of the patients with hepatitis C and *Helicobacter pylori* coinfection received treatment for *H. pylori* prior to the initiation of hepatitis C therapy. Patient 19 failed to take the *H. pylori* medication and has not been treated for hepatitis C. In patient 10, normalization of light chain levels after *H. pylori* treatment and prior to initiation of hepatitis C treatment suggests that the initial light chain elevation was a result of the *H. pylori* infection. The persistence of elevated light chain levels in patients 15, 16, and 18 after *H. pylori* treatment and before hepatitis C treatment raises the possibility that hepatitis C may have contributed to this immune disturbance. Further studies will need to be conducted to further investigate this issue.

The persistence of light chain elevation in 6 out of the 19 patients suggests an ongoing stimulus of the immunoglobulin system which might be the result of either a coinfection with hepatitis C or another comorbid process. Alternatively, in the individuals with persistently elevated light chain levels, the prior infection with *Helicobacter pylori* may have initiated the early stage of a light chain immunoglobulin dysfunction. This potential immunoglobulin system dysfunction may represent the first stage in the development of a monoclonal gammopathy of unknown significance (MGUS), as the stomach, intestines, and associated lymphoid tissues constitute the largest immune organ in the human body, such a hypothesis is worth exploring.

## 5. Summary

(1) What is already known about this subject is as follows.

- The prevalence of *H. pylori* ranges from 30 to 40 percent in the United States.
- *H. pylori* is associated with gastric and duodenal ulcers, gastric adenocarcinoma, and MALT lymphoma.
- Reports of Russell body gastritis in *H. pylori* patients show light chain deposits.

(2) The new findings are as follows.

- This is the first observation of association between *Helicobacter pylori* and an elevation of immunoglobulin free light chains.
- 15/22 patients (68.2%) had at least one light chain elevated during *H. pylori* diagnosis.
- Persistence of light chain elevation in 6/19 patients (31.6%) suggests ongoing stimulus of immunoglobulin system.

(3) How it might impact on clinical practice in forseeable future is as follows:

- give possibility of stratifying *H. pylori* patients into those with abnormal light chain levels and those with normal levels;
- allow clinicians to track patients with persistently elevated light chain levels in what may represent the first stage in the development of a monoclonal gammopathy of unknown significance (MGUS);
- suggest long term follow-up on patients with persistent elevation of light chain levels for a better understanding of the biologic processes involved.

## Figures and Tables

**Table 1:** Patient demographics.

Characteristic	Result
Total patients	22
Sex, %	
Men	68.2
Women	31.8
Age (mean), years	51.9
Age (range), years	30–85
Confirmed *H. Pylori* microbiologic cure*, %	90.9%

*A microbiological cure was confirmed by a positive stool antigen test *via* an enzyme immunoassay by the laboratories at Quest or LabCorp.

**Table 2:** Comorbid conditions.

Diseases	Count
Acquired immunodeficiency syndrome	4
*Chlamydia pneumonia *	2
Coronary artery disease	1
Erythema nodosum	1
Erythrasma	1
Glucose-6-phosphate dehydrogenase deficiency	1
Hepatitis B	3
Hepatitis C	7
Human immunodeficiency virus	2
Human T-lymphotropic virus-I/II	1
Hyperlipidemia	1
Hypertension	3
Idiopathic CD4+ lymphocytopenia	1
Methicillin-resistant *Staphylococcus aureus *	1
Polymyalgia rheumatic	1
Sarcoidosis	1
Schistosomiasis	1
*Strongyloides stercoralis *	4
*Treponema pallidum *	2
Type 2 diabetes mellitus	3

**Table 3:** Treatment regimen for *H. Pylori* patients.

Regimen	Count
Clarithromycin 500 mg po bid, metronidazole 500 mg po tid, omeprazole 20 mg po qid, and bismuth subsalicylate 1 tbsp tid	15
Tetracycline 500 mg po qid, metronidazole 250 mg po tid, omeprazole 20 mg po bid, and bismuth subsalicylate 1 tbsp tid	2
Tetracycline 500 mg po qid, amoxicillin 500 mg po tid, omeprazole 20 mg po bid, and bismuth subsalicylate 1 tbsp tid	1
Tetracycline 500 mg po qid, clarithromycin 500 mg po bid, omeprazole 20 mg po bid, and bismuth subsalicylate 1 tbsp tid	1
Clarithromycin 500 mg po bid, metronidazole 500 mg po tid, lansoprazole 20 mg po bid, and bismuth subsalicylate 1 tbsp tid	1
Clarithromycin 500 mg po bid, amoxicillin 500 mg po tid, lansoprazole 20 mg po bid, and bismuth subsalicylate 1 tbsp tid	1

All regimens initially lasted between 7 and 10 days. Two of the patients were retreated multiple times.

**Table:** (a) Patients with initial normal kappa and lambda light chain levels

Patient	Age	Sex	Comorbid diseases	Time for confirmation of microbiological cure (weeks)	Initial *κ* value (mg/L)	Initial *λ* value (mg/L)
1	30	Female	Hepatitis B	8.1	13.8 (N)	13.3 (N)
2	43	Female	None	4.9	11.7 (N)	12.9 (N)
3	48	Male	Human immunodeficiency virus, *Strongyloides stercoralis*, *Treponema pallidum *	10.3	15.23 (N)	12.36 (N)
4	49	Male	Hepatitis C	6.3	10.6 (N)	14.4 (N)
5	52	Female	Hepatitis B	11.1	17 (N)	24.7 (N)
6	57	Male	Hepatitis C	20.7	15.2 (N)	16.2 (N)
7	59	Female	*Strongyloides stercoralis*, Schistosomiasis	14.1	11.7 (N)	12.6 (N)

**Table:** (b) Patients with elevated kappa and/or lambda light chain levels who experienced normalization of values ^^ ^^

Patient	Age	Sex	Comorbid diseases	Time for confirmation of microbiological Cure (weeks)	Initial *κ* value (mg/L)	Final *κ* value (mg/L)	Normalization of *κ* value	Initial *λ* value (mg/L)	Final *λ* value (mg/L)	Normalization of *λ* value	Time span between initial and final light chain value (weeks)
8	32	Female	Hepatitis B	2	20.2 (A)	16.78 (N)	Yes	15.53 (N)	14.91 (N)	N/A	5
9	42	Male	Acquired immunodeficiency syndrome, human T-lymphotropic virus-I/II, erythrasma	14	19.1 (N)	10 (N)	N/A	27.5 (A)	16.5 (N)	Yes	23.6
10	50	Male	Hepatitis C	4.6	19.7 (A)	16.02 (N)	Yes	31.2 (A)	24.2 (N)	Yes	3.1
11	52	Male	Methicillin-resistant *Staphylococcus aureus*, type 2 diabetes mellitus	129.4	271 (A)	17.4 (N)	Yes	168 (A)	16.9 (N)	Yes	129.4
12	54	Female	Sarcoidosis, idiopathic CD4+ lymphocytopenia, erythema nodosum, hypertension	4.7	28.7 (A)	15.2 (N)	Yes	23.6 (N)	20.9 (N)	N/A	4.7
13	55	Male	*Treponema pallidum*, type 2 diabetes mellitus, hyperlipidemia, hypertension	12.6	22.1 (A)	17.9 (N)	Yes	22.1 (N)	19.6 (N)	N/A	12.6

Patient 8 has a chronic carrier state of hepatitis B surface antigen and a negative viral load. This patient has not received treatment for hepatitis B.

Patient 9 is on HIV medications and had light chain values normalized with *H. pylori* treatment.

Patient 10 received treatment for *H*.* pylori* prior to institution of hepatitis C treatment.

Patient 11 had a methicillin-resistant *Staphylococcus aureus* infection treated with persistently elevated light chain levels that are subsequently normalized after treatment of *H. pylori*.

Patient 13 received treatment for *H. pylori* prior to institution of syphilis treatment.

**Table:** (c) Patients with elevated kappa and/or lambda light chain levels who experienced persistent elevation of values

Patient	Age	Sex	Comorbid diseases	Time for confirmation of microbiological cure (weeks)	Initial *κ* value (mg/L)	Final *κ* value (mg/L)	Normalization of *κ* value	Initial *λ* value (mg/L)	Final *λ* value (mg/L)	Normalization of *λ* value	Time span between initial and final light chain value (weeks)
14	47	Male	Acquired immunodeficiency syndrome	5.9	34.4 (A)	43 (A)	No	49.2 (A)	56.2 (A)	No	5.1
15	52	Male	Hepatitis C, *Chlamydia pneumoniae *	10.6	30.22 (A)	44.74 (A)	No	26.73 (A)	27.07 (A)	No	7.1
16	64	Female	Hepatitis C	11.7	22.9 (A)	23.1 (A)	No	12.3 (N)	11.7 (N)	N/A	4.3
17	51	Male	Acquired immunodeficiency syndrome, glucose-6-phosphate dehydrogenase deficiency, *Strongyloides stercoralis *	34.3	24 (A)	20.8 (A)	No	30 (A)	23.6 (N)	Yes	51.3
18	54	Male	Hepatitis C, hypertension	10.7	21.43 (A)	41.74 (A)	No	31.26 (A)	71.23 (A)	No	11.7
19	71	Male	Hepatitis C, *Strongyloides stercoralis*, type 2 diabetes mellitus	4.3	25.5 (A)	29.1 (A)	No	20.7 (N)	21.1 (N)	N/A	2.7

Patients 15, 16, and 18 who have hepatic C received treatment for *H. pylori* prior to initiation of hepatitis C treatment.

Patient 19 did not receive treatment for *H*.* pylori* and is awaiting further workup for hepatitis C.

**Table:** (d) Patients who were lost to follow-up for repeat light chain values

Patient	Age	Sex	Comorbid diseases	Time for confirmation of microbiological cure (weeks)	Initial *κ* value (mg/L)	Final *κ* value (mg/L)	Normalization of *κ* value	Initial *λ* value (mg/L)	Final *λ* value (mg/L)	Normalization of *λ* value	Time span between initial and final light chain value (weeks)
20	46	Male	*Chlamydia pneumoniae*, human immunodeficiency virus	8.1	340 (A)	Not measured	N/A	195 (A)	Not measured	N/A	N/A
21	48	Male	Acquired immunodeficiency syndrome	Unknown	18 (N)	Not measured	N/A	29.55 (A)	Not measured	N/A	N/A
22	85	Male	Polymyalgia rheumatica, coronary artery disease	Unknown	25.2 (A)	Not measured	N/A	25.6 (N)	Not measured	N/A	N/A
